# Increased gamma band activity for lateral interactions in humans

**DOI:** 10.1371/journal.pone.0187520

**Published:** 2017-12-14

**Authors:** Alon Shapira, Anna Sterkin, Moshe Fried, Oren Yehezkel, Zeev Zalevsky, Uri Polat

**Affiliations:** 1 Nano Photonics Center, the Institute of Nanotechnology and Advanced Materials, Faculty of Engineering, Bar-Ilan University, Ramat-Gan, Israel; 2 Goldschleger Eye Research Institute, Sackler Faculty of Medicine, Tel-Aviv University, Tel-Hashomer, Israel; 3 School of Optometry and Vision Science, Mina & Everard Goodman Faculty of Life Sciences, Bar-Ilan University, Ramat-Gan, Israel; Centre de neuroscience cognitive, FRANCE

## Abstract

Collinear facilitation of contrast sensitivity supported by lateral interactions within primary visual cortex is implicated in contour and object perception, with neural correlates in several frequency bands. Although higher component of the ERP power spectrum, the gamma-band, is postulated to reflect object representation, attention and memory, its neuronal source has been questioned, suggesting it is an artifact reflecting saccadic eye movements. Here we explored the gamma-band activity during collinear facilitation with no saccade-related confounds. We used single-trial spectral analysis of ERP in occipital channels in a time-window of nearly complete saccadic suppression and discarded sporadic trials containing saccades, in order to avoid saccadic artifacts. Although converging evidence suggests that gamma-band oscillations emerge from local excitatory–inhibitory balance involving GABAergic inhibition, here we show activity amplification during facilitatory collinear interactions, presumably dominated by excitations, in the gamma-band 150–350 milliseconds following onset of low near-threshold contrast stimulus. This result highlights the potential role of gamma-band oscillations in neuronal encoding of basic processes in visual perception. Thus, our findings suggest that gamma-band ERP spectrum analysis may serve as a useful and reliable tool for exploring basic perception, both in normal adults and in special populations.

## Introduction

At the earliest stages of visual processing, each neuron responds to stimulation in an isolated region of the visual field, termed classical receptive field (CRF) [1]. Elementary visual signals termed Gabor Patches (GPs) [2], match the CRF profiles in the primary visual cortex [2–7]. Response to GPs presented within the CRF of each neuron can be facilitated (i.e. increasing the sensitivity) or suppressed (i.e. decreasing the sensitivity) by responses to other GPs falling outside the CRF, which, when presented alone, do not activate the neuron. Because these neuronal interactions are mediated by lateral connections within the visual cortex (horizontal connections), they are commonly referred to as lateral interactions. The nature (either facilitation or suppression) and the strength of the lateral interactions are determined by several parameters of the stimulus configuration, such as proximity, similarity, contrast, both inside and outside the CRF [8–14]. Similar effects are found in humans [15–29]. Whereas center-surround effects between the CRF and extra-classical receptive field surround mainly implicate suppression (for review see [30, 31]), collinear facilitation occurs when a near-threshold stimulus inside the CRF is flanked by higher-contrast, collinear (with a similar orientation and positioned along the same axis as their orientation) elements located in surrounding regions of the CRF [11, 20], ideally separated by 3 wavelengths (λ). It is suggested that collinear facilitation constitutes the basic neuronal substrate for object and contour perception in general, and for the neuronal mechanism that is responsible for filling the gaps (filling-in) in contours [22, 29, 31–34]. A recent study mapped the lateral functional connectivity within V1, showing evidence for the psychophysical “association field” for collinear contour perception [35].

Owing to the high temporal resolution of EEG, this technique is widely used for investigating the temporal dynamics of the activity in the human brain. These dynamics entail neuronal oscillations at various different frequencies [36–38]. When networks of neurons are activated by visual stimulation, a synchronous rhythmic activity in the gamma-frequency range (i.e. above 20 Hz, although the exact lower and upper limits vary between different reports, for review see [39]), is observed [40]. This reflects local excitatory-inhibitory interactions that may be modulated by cognitive processes [37, 38, 41–44]. These recordings are usually termed induced gamma-band response. Unlike the phase-locked response (the "evoked" gamma-band response), the transient induced gamma-band response, is characterized by a jittering latency between trials [45].

Gamma oscillations have received much attention in the recent years, especially concerning their role in basic visual processes, such as lateral interactions, as shown by local field potentials [46], as well as higher visual functions, including object and contour representation, attention and memory [40, 47–60]. Gamma oscillations are characterized by a number of properties that suggest a role of a potential neural correlate of high-level processes. For instance, they occur on a time-scale of up to a hundred milliseconds, which matches that of perceptual processes. Moreover, they appear to be synchronized between neural assemblies that are separated in their anatomical locations, which has been postulated as a possible solution to the “binding problem” of consciousness [61]. This makes gamma oscillations relevant to the perceptual binding occurring for low-level visual stimuli that we've proposed earlier to be the underlying mechanism in contrast detection [62]. They were found only in visual cortex in response to specific stimulus properties, such as large size [63, 64], high luminance contrast [65, 66] and regularly-repeating luminance contrasts [67], and recently were shown to reflect feedforward projections in human visual cortex [68]. The latter observation provides support for the recent models of collinear facilitation, which is a part of the feedforward processing and is postulated by the current models to rely on the excitatory–inhibitory balance, with initial fast inhibition followed by a delayed excitation (e.g., [62, 69]). The inhibitory component of collinear facilitation is thus a putative substrate of gamma oscillations.

Understanding the brain function is not the only motivation for deciphering the potential role of cortical gamma oscillations–there is a practical incentive, since many pathological conditions such as epilepsy, phantom perception, and schizophrenia present anomalies of gamma oscillations (for review see [39]).

The most frequently reported finding is a transient power increase a broadband gamma oscillations with a latency of 200 to 300 msec after stimulus onset [54]. Almost all studies report stimulus-driven increase in their magnitude, which is assumed to reflect augmented synchrony, power, or both. Earlier studies in animals suggested that synchronous gamma oscillations reflect the activity of single neurons responding to different fragments of the same object [40, 70]. These studies gave rise to the hypothesis that the neural representation of an object is coded by synchronous gamma-band activity of populations of neurons that encode different parts and features of that object. In visual cortex, gamma oscillations were reported only in response to specific stimulus properties, such as large size [63, 64], high luminance contrast (with no gamma oscillations for color contrast alone despite an equal fMRI response) [65, 66] and regularly repeating luminance contrasts within a specific spatial frequency range [67]. LFP recordings in monkeys using stimuli restricted to the center of the receptive field significantly limit the generation of the gamma drive per se [71]. Another behavioral study examined the effects of the relative phase of gamma frequency flicker (60 Hz) between visual stimuli on the collinear interactions implied that external phase manipulations of gamma frequency do not affect the perceptual integration [72].

The EEG data may often become contaminated by electromyogenic artifacts [51, 73–75]. Artifacts related to involuntary microsaccades were reported to compromise estimates of neuronal activity in the gamma band [51, 76–79]. A study that performed trial-based EEG analysis with simultaneous eye tracking [51] found that transient occipital broadband gamma oscillations with a latency of 300 msec after stimulus onset could be fully accounted for by ocular micro-saccades. Hence, findings of gamma-band oscillations in EEG studies have become somewhat controversial [39].

Studies in humans found enhanced gamma-band response for perceptually-bound image elements, or a "gestalt" perception, relative to perceptually independent elements [47, 48]. Here we aimed to explore the gamma-band activity during collinear facilitation in the Event-Related Potentials (ERPs), suggested as a low-level stage of visual processing supporting contour and object perceptual binding, with no contamination of saccade-related confounds in occipital channels. The analyzed time interval was 0–400 msec after the stimulus onset, corresponding to the time-window of collinear facilitation processing [80], by the end of which the processing of the feedforward sweep for grouping of contour elements is accomplished [81] and before the microsaccadic artifact. Simultaneous high-resolution eye tracking confirmed suppression of saccadic eye movements in this time window, in order to avoid electromyogenic artifacts suggested for longer latencies [51]. ERPs are a widely-used measure of “brain response that is the direct result of a specific sensory, cognitive, or motor event”. Here we applied an approach of the ERP data processing that is different from the largely used standard, however is widely accepted in signal processing [82].

## Methods

### Subjects

Event-related brain potentials (ERPs) were recorded in 4 volunteers (1 female, aged 40±14 years, mean ± STD), 3 naive and one co-author (M.F.) with normal or corrected-to-normal vision in both eyes and had no history of neurological or psychiatric illness. The Human Research Committee at the Sheba Medical Center approved the study. Informed written consent was obtained from all subjects. All experimental protocols were performed in accordance with the guidelines provided by the committee approving the experiments.

### Stimuli

**Fig 1:**
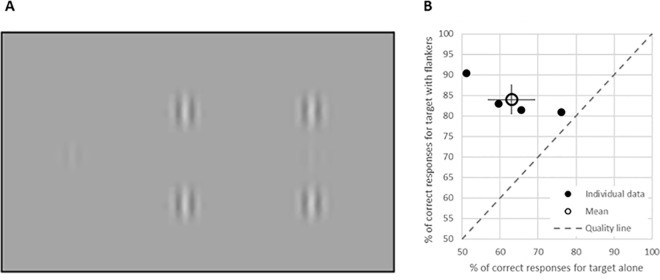
Stimuli and behavioral results. (A) The stimuli contained either a target GP presented in isolation at a contrast of 8%, which is near the detection threshold (left; contrast amplified for presentation), collinear flankers (middle; 2 collinear GPs at a contrast of 40%, each separated by 3λ from the center of the screen, or a target with collinear flankers, each separated by 3λ from the target (right, termed lateral masking (LM)). (B) Percent of correct responses for trials with target presented in isolation (abscissa) and with flankers (ordinate). Solid symbols, individual data of the 4 subjects; open symbol, mean; error bars, STD; dotted gray line, quality line.

The stimuli were vertically-oriented localized gray-level gratings (GPs) with spatial frequency of 6 cycles per degree (wavelength, λ) and equal distribution (standard deviation (STD) one σ, allowing a minimum of 2 cycles in the GP), modulated from a background luminance of 40 cd·m^-2^ (Fig 1A). A Philips 107P color monitor was used for stimulus presentation in a dark room, with a resolution of1024 x 768 pixels, a refresh rate of 100 Hz, an effective size of 32 × 24 cm, subtending a visual angle of 9.1 × 12.1 degrees at a viewing distance of 100 cm. Gamma correction was applied.

### Paradigm

ERPs were recorded for the following conditions: 1) a foveal vertically oriented GP target presented alone at a contrast of 8% (at or very near the detection threshold), i.e., the “Target” condition; 2) two collinear GPs separated by 6 wavelengths (λ), equally placed above and below the center of the display, at a contrast of 40%, i.e., the “Flankers”condition; 3) target with flankers each separated by 3λ, also termed lateral masking (LM) collinear interactions (Fig 1A), i.e., the “Lateral” condition, and 4) no stimuli at all, i.e., the “Nothing” condition. Contrast detection facilitation, which is the phenomenon of interest here, was shown earlier to preferentially occur for collinear as opposed to orthogonal flankers [11, 20]. Under all conditions, the target GP was present in half of the trials and the task was to report the detection of the target using a standard computer mouse. A Yes/No paradigm was used: response was required in each trial–left button for a "yes" and right button for "no" response, as fast as possible, with no delay. No feedback was provided. Stimuli were presented for 50 msec, every 2000 msec. All conditions were mixed in a random order, 100 trials per condition. Participants were instructed to maintain their fixation on a 0.1-degree static black dot in the center of the screen and to avoid eye movements during and between the trials. Blinks and high amplitude eye movements (see below) were discarded.

### Eye tracking data recording and analysis

Eye movements were recorded for the dominant eye only using an EyeLink® 1000 desktop model, SR-Research. Before each recording, the system was calibrated in order to obtain an accurate gaze position. The head position was secured using a chin-rest. Eye tracking data were sampled at 500Hz and smoothed by a low-pass filter with a cut-off frequency of 120Hz.

**Fig 2:**
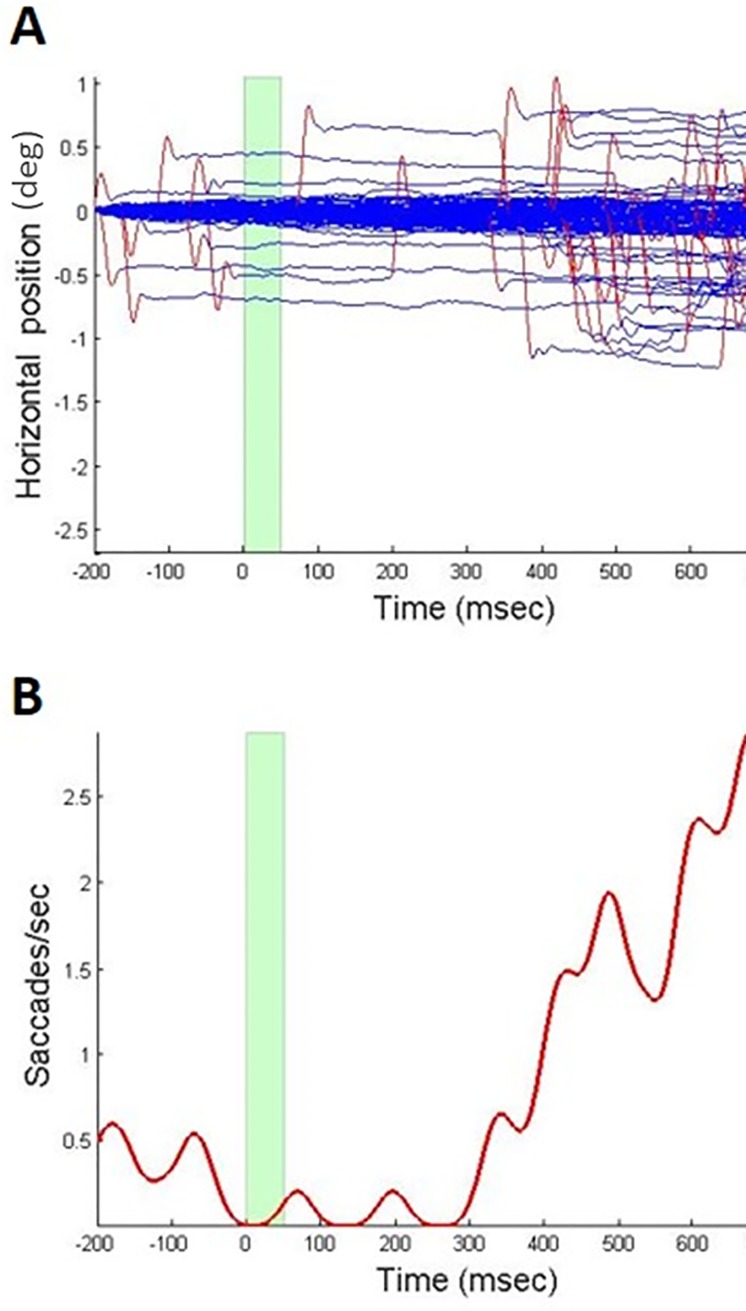
Eye tracking data example. (A) Horizontal position of the dominant eye, relative to the ‘position relative to the gaze position at time of -200 msec is shown for a representative subject BH (ordinate) as a function of time (abscissa) relative to stimulus onset, across all conditions, for a total of 400 trials. The light green bar shows target durati on. Subject was instructed to fixate in the center of the screen throughout the whole experiment. Microsaccades are marked in red. (B) Corresponding microsaccade rate for data shown in A.

Blinks were detected as periods of no data. Microsaccades were detected by an algorithm developed by M.F., as was reported earlier [83, 84]. Data samples representing eye movement for at least 6 msec in the same direction (with a 30-degrees window), with the minimum velocity, checked with each sample, above 10 degrees per second, a peak velocity above 18 degrees per second, and a saccade amplitude above 0.1 degrees, were detected (Fig 2). Saccades with amplitudes above 2 degrees were discarded. Dynamic overshoots, i.e. lower-amplitude microsaccades that occur immediately after a microsaccade in the opposite direction, were counted together with the main microsaccade. Blinks, including 20 msec before and after each blink, were discarded.

Microsaccade and blink rate modulation functions throughout the trials, time-locked to stimulus onset, were computed as follows: In each trial, blinks (or microsaccades) were summed as Gaussians with the center at the time of onset and with a sigma of 20 msec to obtain a rate modulation function per trial. This analysis was performed in the time-window between -200 to 800 msec. These rate modulations were averaged within subjects, and then averaged across subjects. A similar method was used successfully earlier [83, 84], applied here for both microsaccades and blinks.

### ERP data recording and analysis

The ERP recording was performed using the setup similar to the ones published earlier [85]. The EEG was from a cruciform array of 5 channels centered at a midline occipital channel (Oz), spaced by 3 cm, and referenced to the midline frontal channel, Fz (sampling rate of 1032.5 Hz, filtered between 0.1–100 Hz, amplified by 50000 using Grass Model 12 amplifiers). The average ERPs were computed over 2000 msec period per trial, for 100 trials per condition. The recorded data were cut into segments of 4096 samples, starting 250 msec before stimulus onset of each trial. Trials with saccades within the first 400 msec from stimulus onset were disregarded (see Table 1 for the prevalence of trails without saccades). The segments were then sorted into 8 groups according to the possible combinations of trial type and response (also termed "Latin Square", Table 1). The following analysis focused only the conditions *Lateral Hit*, *Flankers Correct Reject* and *Target Hit* (in bold).

**Table 1:** Prevalence of the saccades in the 0–400 msec window in the 8 possible combinations of trial type and response ("Latin Square"), averaged for the 4 subjects (mean ± STD).

Trial type & Response	Percent of trials without saccades from the total number of trials
Lateral Hit	84.4 ± 4.1%
Lateral Miss	74.2 ± 7.0%
Flankers Correct Reject	83.9 ± 6.4%
Flankers False Alarm	72.1 ± 17.9%
Target Hit	80.6 ± 12.8%
Target Miss	65.9 ± 23.6%
Nothing Correct Reject	66.0 ± 21.9%
Nothing False Alarm	81.1 ± 10.4%

For each one of the 4 subjects, we calculated the power spectrum (spectrogram) of 3 conditions: *Lateral Hit* (LH), *Flankers Correct Reject* (FC) and *Target Hit* (TH).

The general formula reads as follows:

$$X(\tau ,\omega )={\left|\int_{-\infty }^{\infty }x(t)W(t-\tau )e^{-i\omega t}dt\right|}^{2},$$ (1)

where *x*(*t*) is the time dependent signal, *ω* is the frequency, *t*, *τ* represent the time and

*W*(*t*) is a windowing function. In the discrete case, Eq (1) becomes

$$X[t,f]={\left|\sum_{n=1}^{N}x[n]W[n-t]e^{\frac{-2\pi i}{N}(t-1)(f-1)}\right|}^{2}.$$ (2)

In practice, the calculation was performed by using the MATLAB command [~,f,t,X] = spectrogram(x,hann(L),L-1,N,Fs), where x is the trial vector, consisting of 4096 points (N) starting 250 msec prior to stimulus onset, samples in frequency of Fs = 1302.5 Hz. The windowing function had width of L = 256. *X* is the resulting spectrogram.

Here, *X* is a matrix whose rows are time dependent signals for each frequency. Thus, *t*, *f* are the discrete time and space variables, and *N* is the length of the signal vector *x*. The *Hanning* windowing function *W*(*n*) is defined as

$$W(n)=\frac{1}{2}\left(1-\cos\frac{2\pi n}{L-1}\right),$$ (3)

where *L* is the width of the window. For the following calculation, we used *N* = 4096, *L* = 256. Each spectrogram was normalized via

$$X^{o}=\frac{X-B}{B_{SD}},$$ (4)

where *X*^*o*^ is a normalized spectrogram of a single trail. The matrix *B* is the corresponding *baseline matrix* for each *X* separately, which is defined as:

$$B=[\begin{array}{ccc} b(f) & b(f) & \ldots \end{array}],$$ (5)

where all columns are identical and *b*(*f*) is the average over time of the last 500ms of a given spectrogram (which is the pre-stimulus baseline for the consecutive stimulus):

$$b(f)=\frac{1}{t_{2000}-t_{1500}}\sum_{t=t_{1500}}^{t_{2000}}X[t,f].$$ (6)

In (6), *t*_1500_, *t*_2000_ indicate the discrete time points where *t* ≈ 1500 msec and *t* ≈ 2000 msec, respectively. *B*_*SD*_ is the standard deviation matrix of all *B* matrices per subject [86]. All *X*^*o*^’s of each condition (LH, FC and TH) for a given subject were averaged, obtaining 3 averaged spectrograms for each one of the 4 subjects and which are denoted as: $A_{LH}^{i},A_{FC}^{i},A_{TH}^{i}$, where *i* ∈ {1,2,3,4}.

Next, a linear prediction (LP) of no lateral interaction for the LH condition type was calculated as the sum of the FC and TH averaged spectrograms (i.e., for target presented in isolation and for flankers presented in isolation, with correct responses):

$$A_{LP}^{i}=A_{FC}^{i}+A_{TH}^{i}.$$ (7)

Since low frequencies are several orders of magnitudes above gamma frequency, patterns are easier to be observed in log-scale (dB). In attempt to eliminate negative elements, we have subtracted the minimum element, $m_{A}^{i}$, of both $A_{LP}^{i}$ and $A_{LH}^{i}$ before presenting them in log-scale:

$${\tilde{A}}_{LH}^{i}=10\;\log_{10}\left(A_{LH}^{i}-m_{A}^{i}\right),$$ (8)

$${\tilde{A}}_{LP}^{i}=10\;\log_{10}\left(A_{LP}^{i}-m_{A}^{i}\right),$$ (9)

The number $m_{A}^{i}$ is calculated in MATLAB by m = min(min([A_LH A_LP]))-1e-4. The small subtraction to m is in order to avoid zero elements after subtraction, that would give -∞ in log-scale. The amplification of LH condition over the linear prediction is simply the difference:

$$D^{i}={\tilde{A}}_{LH}^{i}-{\tilde{A}}_{LP}^{i}.$$ (10)

**Fig 3:**
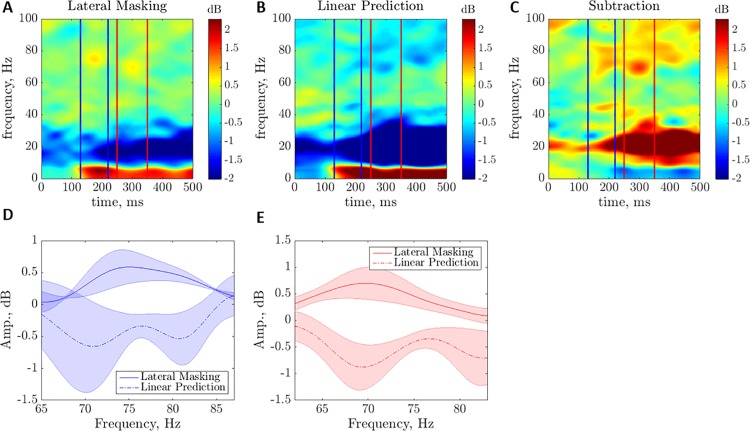
Linear interactions demonstrated in the ERP power spectrum. Average amplification spectrograms of (A) lateral masking with correct responses, (B) linear prediction of no interaction and (C) their difference, within the two peaks in the gamma-band. (D) Mean magnitude vs. frequency of (A) and (B) for the first peak, averaged for the 4 subjects. Lateral Masking, solid; Linear Prediction, dashed; shaded colors, SE. (E) As in (D), for the second peak.

The matrices *D*^*i*^ were inspected for amplification in the gamma band (30–80 Hz) along the 500 msec after stimulus onset. Two peaks were found to be bounded, in each one of the subjects, in the following time windows: first at 150–250 msec (*t*_1_) and 70–85 Hz, and second at 250–350 msec (*t*_2_) and 60–75 Hz (the spectrograms per subject are presented in the Supplementary Information). *t*_1_ and *t*_2_ correspond to maximal power point of each one of the two peaks. Next, calculating the zero-mean spectrograms of ${\tilde{A}}_{LH}^{i}$ and ${\tilde{A}}_{LP}^{i}$ allows us to present LH and LP in the same scale with the spectrogram *D*^*i*^ (Fig 3A–3C):

$$Z_{LH}^{i}={\tilde{A}}_{LH}^{i}-M_{Z}^{i},$$ (11)

$$Z_{LP}^{i}={\tilde{A}}_{LP}^{i}-M_{Z}^{i}$$ (12)

The number $M_{Z}^{i}$ is calculated as the average of all elements of both ${\tilde{A}}_{LH}^{i}$ and ${\tilde{A}}_{LP}^{i}$. We follow by defining the frequency profiles for each peak:

$${p_{LH}^{i}}_{1}[f]=Z_{LH}^{i}[t_{1},f],\;{p_{LP}^{i}}_{1}[f]=Z_{LP}^{i}[t_{1},f],$$ (13)

$${p_{LH}^{i}}_{2}[f]=Z_{LH}^{i}[t_{2},f],\;{p_{LP}^{i}}_{2}[f]=Z_{LP}^{i}[t_{2},f].$$ (14)

The average profiles are depicted in Fig 3D (for peak 1) and 3E (for peak 2) (the profiles per subject are presented in the Supplementary Information).

### Statistical analysis

Behavioral data yielded an accuracy measure (% of correct responses; a single measure per subject) that compared using the Wilcoxon Signed-Rank Test (W test) for paired data sets, which does not require the assumption of normal distributions that is impossible for limited sample sizes. The ERP data were compared using the W test for the LH and LP conditions, per peak. To that end, the summed magnitude over the profiles within the peak coordinates (i.e., the two minimum points surrounding each peak in the curve of the subtraction (see Eq (10)); peak 1: between 60.4 and 88 Hz; peak 2: between 59.2 and 77.9 Hz) was calculated, per subject (peak 1: 26.4 ± 14, mean ± STD for LH and -25.6 ± 55 for LP; peak 2: 31.1 ± 21 for LH and -35.1 ± 38 for LP).

## Results

We first confirmed the experimental conditions evoke collinear facilitation, manifested as a higher accuracy during detection of GP targets with near-threshold contrast when presented with flankers compared to isolated presentation (Fig 1A). To that end, the percent of correct responses for trials with target presented in isolation was plotted against the percent of correct responses for trials with flankers (Fig 1B), only for trials without microsaccades in the 0–400 msec time window after the stimulus onset. In concert with the earlier reports [20, 22, 32, 33], there was a significant facilitation of 21% on average in contrast detection (from 63 to 84%, W test: p = 0.034).

Fig 2A depicts the time-course of the eye movements throughout the trial, with a clear microsaccade suppression during the 0–400 msec time-window (Fig 2B), similar to the earlier reported saccadic suppression for anticipated stimuli [83, 84].

In the spectral analysis of LM trials with correct responses (*Lateral Hit*, Fig 3A), two peaks in the gamma frequency range were observed, in each one of the subjects, quantified within the following coordinates: first at 70.5±7.8 (mean±STD) Hz and 200.7±34.1 msec, and second at 68.4±3.3 Hz and 298.9±6.1 msec. These peaks do not exist in the prediction of no lateral interaction in the LM (*Linear Prediction*, calculated as a sum of data produced for target presented in isolation and for flankers presented in isolation, with correct responses [80]; Fig 3B). The subtraction of the mean magnitude of the *Linear Prediction* from that of the *Lateral Hit*, averaged for the 4 subjects (Fig 3C), shows an amplification of up to 1.8 dB for the LM over prediction of no interaction (1.5±1.2 dB for the first peak and 1.8±0.6dB for the second peak). This significant difference is depicted per peak in Fig 3D and 3E (W test p = 0.014 for the first peak and 0.001 for the second peak).

**Fig 4:**
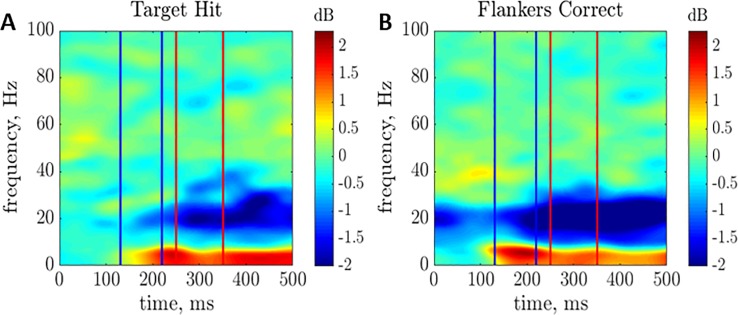
Linear prediction components. Average amplification spectrograms of (A) Target Hit (TH) and (B) Flankers Correct Reject (FC) conditions.

Fig 4 shows the averaged spectrograms for the Target Hit (TH) and Flankers Correct Reject (FC) conditions (i.e., for target presented in isolation and for flankers presented in isolation, with correct responses).

## Discussion

Here we show narrowband (or “bump”) gamma oscillation evoked by small localized stimuli of low near-threshold contrast. This is in contrast with broad-band gamma oscillations, starting above 20 Hz [40, 70] and extending up to over 160 Hz [63, 64], largely transient subsequent to stimulus onset and sometimes proposed to simply indicate spectral leakage from multi-unit activity [87]. Our results show that lateral interactions with low contrast target and small stimulus size have a correlate in the gamma-band ERP spectrum in trials with no saccades. Studies in sensory domains other than the visual have not pointed out narrowband gamma oscillations (for review see [39]). Gamma oscillations were found in visual cortex only, evoked by specific stimulus properties, like large size [63, 64], high luminance contrast (without gamma oscillations for color contrast alone despite an equal fMRI response) [65, 66] and regularly-repeating luminance contrasts within a specific range of spatial frequencies [67].

It was suggested that collinear interactions mediate object and contour perception [22, 31–34], simulated as a network of excitation and inhibition [62, 88]. Converging evidence suggests that gamma-band oscillations emerge from local excitatory–inhibitory balance [38]. Within each oscillatory cycle, excitatory neurons evoke GABAergic interneurons, in turn suppressing local excitation. Another likely contribution to the generation of local gamma-band oscillations is ascribed to inhibitory interneurons that shape local representations [89, 90]. The central frequency of narrowband visual gamma is determined by the local concentration of the GABA inhibitory neurotransmitter and is inversely correlated to the fMRI response to visual stimulation in humans [91]. Furthermore, it is correlated with the behavioral measurements in visual orientation discrimination tasks [92]. Therefore, stimulus selection is one of the potential applications of visual narrowband gamma, with yet undetermined exact nature of that application. Likewise, the magnitude of visual stimulus-induced narrowband gamma oscillations in middle occipital gyrus adjacent to a stimulus change predicts the speed of change detection [93]. In the context of gamma oscillations, inhibitory interneurons may also have a role as a “temporal pacemaker”, leading to coordinated behavior in a group of neurons (for a review, see [94]).

Higher gamma amplitude could be potentially related to a stronger sensory representation and thus a faster change detection, reflecting a positive role. Conversely, it could attenuate irrelevant stimulus features and thus a faster identification of changes in relevant features, reflecting an inhibitory role. On one hand, increase in stimulus contrast monotonically increases gamma oscillations [46] and reduces collinear facilitation, including in single unit recording [11, 19]. However, on the other hand, both these results could emerge in an inhibition-stabilized network [95]. Our findings show gamma amplification during facilitatory collinear interactions for low near-threshold stimulus contrast, though probably dominated by excitations [13], suggesting a positive role. Its power is probably much lower than the maximum observed for high contrast stimuli. The co-occurrence between collinear facilitation of contrast detection and narrowband visual gamma amplification is particularly noteworthy in light of earlier studies showing a robust reduction in collinear facilitation in patients with depression [96] and in normal subjects under the effect of enhanced GABAergic inhibition by *benzodiazepine* [97].

It is widely acknowledged that whereas theta and gamma oscillations predominate in the feedforward processes, beta oscillations predominate in the feedback processes (for a recent review, see [98]). Predictive coding models predict that feedback cortico-cortical connections transport prediction signals at slower time-scales (e.g. beta) compared to feedforward connections that convey prediction error signals at faster time scales (e.g. gamma). Bauer and colleagues [99] have shown that surprise was tracked by attention-dependent gamma-band oscillations. These finding suggests that prediction errors are mediated by gamma-band oscillations. Furthermore, Van Kerkoerle and colleagues studied the propagation of alpha and gamma oscillations by inducing oscillations with microstimulation and pharmacological manipulation and showed that whereas gamma oscillations underlie the feedforward processing, alpha oscillations underlie the feedback processing [100].

Our aim was to explore the gamma-band ERP responses during collinear facilitation with no saccade-related confounds. Our results show that lateral interactions in the basic levels of visual processing co-occur with amplification in the gamma-band ERP spectrum in trials with no saccades, proving further insights onto the previously suggested potential role of these high-frequency oscillations in neuronal encoding of basic processes in visual perception. The observed gamma amplification during lateral interactions suggest a positive role. Our findings suggest that gamma-band ERP spectrum analysis may serve as a useful and reliable tool for exploring basic perception, both in normal adults and in special populations, such as infants, autistic individuals or patients with schizophrenia.

## Supporting information

S1 File A) Spectrograms per subject for the lateral masking condition with correct responses (Lateral Hit). B) Spectrograms per subject for the linear prediction. C) Spectrograms per subject for the subtraction between Lateral Masking with correct responses (Lateral Hit) and linear prediction. D) Profiles per subject, including the values of the peaks.
(PDF)

S2 File Raw data. Raw ERP, eye tracking and behavioral data per subject.
(RAR)

## References

[1] Hubel DH, Wiesel TN. Receptive fields of single neurones in the cat's striate cortex. J Physiol. 1959;148:574–91. Epub 1959/10/01. 14403679 10.1113/jphysiol.1959.sp006308 PMC1363130

[2] Gabor D. Theory of communication. Journal of the Instifufe of Electrical Engineers London,. 1946;93:429–57.

[3] Marčelja S. Mathematical description of the responses of simple cortical cells*. JOSA. 1980;70(11):1297–300. 10.1364/josa.70.001297 7463179

[4] Turner MR. Texture discrimination by Gabor functions. Biol Cybern. 1986;55:71–82. 3801538 10.1007/BF00341922

[5] Caelli T, Moraglia G. On the detection of Gabor signals and discrimination of Gabor textures. Vision Res 1985;25. 10.1016/0042-6989(85)90173-7 4024467

[6] Daugman JD. Image analysis and compact coding by oriented 2-D Gabor primitives. SPIE Process 1987;758:19–30

[7] Beck J, Sutter A, Ivry R. Spatial frequency channels and perceptual grouping in texture segregation. Comput Vision, Graph Image Proc 1987;37:299–325.

[8] Crook JM, Engelmann R, Lowel S. GABA-inactivation attenuates colinear facilitation in cat primary visual cortex. Exp Brain Res. 2002;143(3):295–302. 10.1007/s00221-002-1007-y 11889507

[9] Kapadia MK, Ito M, Gilbert CD, Westheimer G. Improvement in visual sensitivity by changes in local context: parallel studies in human observers and in V1 of alert monkeys. Neuron. 1995;15(4):843–56. 7576633 10.1016/0896-6273(95)90175-2

[10] Mizobe K, Polat U, Pettet MW, Kasamatsu T. Facilitation and suppression of single striate-cell activity by spatially discrete pattern stimuli presented beyond the receptive field. Vis Neurosci. 2001;18(3):377–91. 11497414 10.1017/s0952523801183045

[11] Polat U, Mizobe K, Pettet MW, Kasamatsu T, Norcia AM. Collinear stimuli regulate visual responses depending on cell's contrast threshold. Nature. 1998;391(6667):580–4. 10.1038/35372 9468134

[12] Series P, Lorenceau J, Fregnac Y. The "silent" surround of V1 receptive fields: theory and experiments. J Physiol Paris. 2003;97(4–6):453–74. 10.1016/j.jphysparis.2004.01.023 . 15242657

[13] Kasamatsu T, Miller R, Zhu Z, Chang M, Ishida Y. Collinear facilitation is independent of receptive-field expansion at low contrast. Experimental Brain Research. 2010;201(3):453–65. Epub 2009/11/06. 10.1007/s00221-009-2057-1 . 19888567 PMC3252032

[14] Chen CC, Kasamatsu T, Polat U, Norcia AM. Contrast response characteristics of long-range lateral interactions in cat striate cortex. Neuroreport. 2001;12(4):655–61. 11277558 10.1097/00001756-200103260-00008

[15] Bonneh Y, Sagi D. Configuration saliency revealed in short duration binocular rivalry. Vision Res. 1999;39(2):271–81. 10326135 10.1016/s0042-6989(98)00112-6

[16] Ellenbogen T, Polat U, Spitzer H. Chromatic collinear facilitation, further evidence for chromatic form perception. Spat Vis. 2006;19(6):547–68. . 17278527 10.1163/156856806779194062

[17] Cass J, Alais D. The mechanisms of collinear integration. J Vis. 2006;6(9):915–22. 10.1167/6.9.5 . 17083284

[18] Cass JR, Spehar B. Dynamics of collinear contrast facilitation are consistent with long-range horizontal striate transmission. Vision Res. 2005;45(21):2728–39. 10.1016/j.visres.2005.03.010 . 16038960

[19] Polat U, Norcia AM. Neurophysiological evidence for contrast dependent long-range facilitation and suppression in the human visual cortex. Vision Res. 1996;36(14):2099–109. 8776476 10.1016/0042-6989(95)00281-2

[20] Polat U, Sagi D. Lateral interactions between spatial channels: suppression and facilitation revealed by lateral masking experiments. Vision Res. 1993;33(7):993–9. 8506641 10.1016/0042-6989(93)90081-7

[21] Polat U, Sagi D. Spatial interactions in human vision: from near to far via experience- dependent cascades of connections. Proc Natl Acad Sci U S A. 1994;91(4):1206–9. 8108388 10.1073/pnas.91.4.1206 PMC43125

[22] Polat U, Sagi D. The architecture of perceptual spatial interactions. Vision Res. 1994;34(1):73–8. 8116270 10.1016/0042-6989(94)90258-5

[23] Polat U, Sagi D. Temporal asymmetry of collinear lateral interactions. Vision Res. 2006;46(6–7):953–60. 10.1016/j.visres.2005.09.031 . 16274724

[24] Shani R, Sagi D. Psychometric curves of lateral facilitation. Spat Vis. 2006;19(5):413–26. . 17131648 10.1163/156856806778457386

[25] Tanaka Y, Sagi D. Long-lasting, long-range detection facilitation. Vision Res. 1998;38(17):2591–9. . 12116705 10.1016/s0042-6989(97)00465-3

[26] Solomon JA, Morgan MJ. Facilitation from collinear flanks is cancelled by non-collinear flanks. Vision Res. 2000;40(3):279–86. 10793901 10.1016/s0275-5408(99)00059-9

[27] Woods RL, Nugent AK, Peli E. Lateral interactions: size does matter. Vision Res. 2002;42(6):733–45. 11888539 10.1016/s0042-6989(01)00313-3

[28] Herzog MH, Fahle M. Effects of grouping in contextual modulation. Nature. 2002;415(6870):433–6. 10.1038/415433a . 11807555

[29] Lev M, Polat U. Collinear facilitation and suppression at the periphery. Vision Res. 2011;51(23–24):2488–98. Epub 2011/11/01. 10.1016/j.visres.2011.10.008 . 22037360

[30] Angelucci A, Bressloff PC. Contribution of feedforward, lateral and feedback connections to the classical receptive field center and extra-classical receptive field surround of primate V1 neurons. In: Martinez-Conde S. SLMLMMJMA, Tse PU, editors. Progress in Brain Research. Volume 154, Part A: Elsevier; 2006 p. 93–120. 17010705 10.1016/S0079-6123(06)54005-1

[31] Gilbert CD. Adult cortical dynamics. Physiological Reviews. 1998;78(2):467–85. 9562036 10.1152/physrev.1998.78.2.467

[32] Polat U, Bonneh Y. Collinear interactions and contour integration. Spat Vis. 2000;13(4):393–401. 11310533 10.1163/156856800741270

[33] Polat U, Sagi D. The relationship between the subjective and objective aspects of visual filling-in. Vision Res. 2007 10.1016/j.visres.2007.06.007 . 17655907

[34] Meirovithz E, Ayzenshtat I, Bonneh YS, Itzhack R, Werner-Reiss U, Slovin H. Population response to contextual influences in the primary visual cortex. Cerebral Cortex. 2010;20(6):1293–304. 10.1093/cercor/bhp191 19759123

[35] Gerard-Mercier F, Carelli PV, Pananceau M, Troncoso XG, Frégnac Y. Synaptic correlates of low-level perception in V1. The Journal of Neuroscience. 2016;36(14):3925–42. 10.1523/JNEUROSCI.4492-15.2016 27053201 PMC6705520

[36] Wang X-J. Neurophysiological and Computational Principles of Cortical Rhythms in Cognition. Physiological Reviews. 2010;90(3):1195–268. 10.1152/physrev.00035.2008. PMC2923921. 20664082 PMC2923921

[37] Siegel M, Donner TH, Engel AK. Spectral fingerprints of large-scale neuronal interactions. Nat Rev Neurosci. 2012;13(2):121–34. 10.1038/nrn3137 22233726

[38] Donner TH, Siegel M. A framework for local cortical oscillation patterns. Trends in Cognitive Sciences. 2011;15(5):191–9. 10.1016/j.tics.2011.03.007 21481630

[39] Sedley W, Cunningham MO. Do cortical gamma oscillations promote or suppress perception? An under-asked question with an over-assumed answer. Frontiers in Human Neuroscience. 2013;7 10.3389/fnhum.2013.00595 24065913 PMC3778316

[40] Gray CM, Konig P, Engel AK, Singer W. Oscillatory responses in cat visual cortex exhibit inter-columnar synchronization which reflects global stimulus properties. Nature. 1989;338(6213):334–7. 10.1038/338334a0 2922061

[41] Hasenstaub A, Shu Y, Haider B, Kraushaar U, Duque A, McCormick DA. Inhibitory Postsynaptic Potentials Carry Synchronized Frequency Information in Active Cortical Networks. Neuron. 2005;47(3):423–35. 10.1016/j.neuron.2005.06.016 16055065

[42] Bartos M, Vida I, Jonas P. Synaptic mechanisms of synchronized gamma oscillations in inhibitory interneuron networks. Nat Rev Neurosci. 2007;8(1):45–56. 10.1038/nrn2044 17180162

[43] Cardin JA, Carlen M, Meletis K, Knoblich U, Zhang F, Deisseroth K, et al Driving fast-spiking cells induces gamma rhythm and controls sensory responses. Nature. 2009;459(7247):663–7. http://www.nature.com/nature/journal/v459/n7247/suppinfo/nature08002_S1.html. 10.1038/nature08002 19396156 PMC3655711

[44] Fries P. Neuronal Gamma-Band Synchronization as a Fundamental Process in Cortical Computation. Annual Review of Neuroscience. 2009;32(1):209–24. 10.1146/annurev.neuro.051508.135603 19400723

[45] Pantev C. Evoked and induced gamma-band activity of the human cortex. Brain Topography. 1995;7(4):321–30. 10.1007/bf01195258 7577330

[46] Henrie JA, Shapley R. LFP power spectra in V1 cortex: the graded effect of stimulus contrast. Journal of Neurophysiology. 2005;94(1):479–90. 10.1152/jn.00919.2004 15703230

[47] Lutzenberger W, Pulvermu¨ ller F, Elbert T, Birbaumer N. Visual-Stimulation Alters Local 40-Hz Responses in Humans—an Eeg Study. Neurosci Lett 1995;183:39–42. 7746482 10.1016/0304-3940(94)11109-v

[48] Tallon-Baudry C, Bertrand O, Delpuech C, Pernier J. Stimulus specificity of phase-locked and non-phase-locked 40 Hz visual responses in human. J Neurosci. 1996;16(13):4240–9. Epub 1996/07/01. . 8753885 10.1523/JNEUROSCI.16-13-04240.1996 PMC6579008

[49] Gruber T, Muller MM. Oscillatory brain activity dissociates between associative stimulus content in a repetition priming task in the human EEG. Cereb Cortex. 2005;15(1):109–16. Epub 2004/07/09. 10.1093/cercor/bhh113 . 15238442

[50] Zion-Golumbic E, Bentin S. Dissociated neural mechanisms for face detection and configural encoding: evidence from N170 and induced gamma-band oscillation effects. Cereb Cortex. 2007;17(8):1741–9. Epub 2006/10/26. 10.1093/cercor/bhl100 . 17062635

[51] Yuval-Greenberg S, Tomer O, Keren AS, Nelken I, Deouell LY. Transient Induced Gamma-Band Response in EEG as a Manifestation of Miniature Saccades. Neuron. 2008;58(3):429–41. 10.1016/j.neuron.2008.03.027 18466752

[52] Rodriguez E, George N, Lachaux J-P, Martinerie J, Renault B, Varela FJ. Perception's shadow: long-distance synchronization of human brain activity. Nature. 1999;397(6718):430–3. 10.1038/17120 9989408

[53] Tallon-Baudry C, Bertrand O, Delpuech C, Permier J. Oscillatory gamma-band (30–70 Hz) activity induced by a visual search task in humans. J Neurosci. 1997;17(2):722–34. Epub 1997/01/15. . 8987794 10.1523/JNEUROSCI.17-02-00722.1997 PMC6573221

[54] Tallon-Baudry C, Bertrand O. Oscillatory gamma activity in humans and its role in object representation. Trends in Cognitive Sciences. 1999;3(4):151–62. 10.1016/S1364-6613(99)01299-1. 10322469

[55] Gruber T, Tsivilis D, Montaldi D, Müller MM. Induced gamma band responses: an early marker of memory encoding and retrieval. Neuroreport. 2004;15(11):1837–41. 00001756-200408060-00030. 15257158 10.1097/01.wnr.0000137077.26010.12

[56] Herrmann CS, Munk MH, Engel AK. Cognitive functions of gamma-band activity: memory match and utilization. Trends Cogn Sci. 2004;8(8):347–55. Epub 2004/09/01. 10.1016/j.tics.2004.06.006 . 15335461

[57] Keil A, Gruber T, Muller MM. Functional correlates of macroscopic high-frequency brain activity in the human visual system. Neurosci Biobehav Rev. 2001;25(6):527–34. Epub 2001/10/12. . 11595272 10.1016/s0149-7634(01)00031-8

[58] Varela F, Lachaux JP, Rodriguez E, Martinerie J. The brainweb: phase synchronization and large-scale integration. Nat Rev Neurosci. 2001;2(4):229–39. Epub 2001/04/03. 10.1038/35067550 . 11283746

[59] Lachaux J-P, George N, Tallon-Baudry C, Martinerie J, Hugueville L, Minotti L, et al The many faces of the gamma band response to complex visual stimuli. Neuroimage. 2005;25(2):491–501. 10.1016/j.neuroimage.2004.11.052. 10.1016/j.neuroimage.2004.11.052 15784428

[60] Osipova D, Takashima A, Oostenveld R, Fernández G, Maris E, Jensen O. Theta and Gamma Oscillations Predict Encoding and Retrieval of Declarative Memory. The Journal of Neuroscience. 2006;26(28):7523–31. 10.1523/JNEUROSCI.1948-06.2006 16837600 PMC6674196

[61] Singer W, Gray CM. Visual Feature Integration and the Temporal Correlation Hypothesis. Annual Review of Neuroscience. 1995;18(1):555–86. 10.1146/annurev.ne.18.030195.003011 . 7605074

[62] Sterkin A, Sterkin A, Polat U. Response similarity as a basis for perceptual binding. J Vis. 2008;8(7):1–12 10.1167/8.7.17 19146250

[63] Jia X, Smith MA, Kohn A. Stimulus Selectivity and Spatial Coherence of Gamma Components of the Local Field Potential. The Journal of Neuroscience. 2011;31(25):9390–403. 10.1523/JNEUROSCI.0645-11.2011 21697389 PMC3133446

[64] Ray S, Maunsell JH. Different origins of gamma rhythm and high-gamma activity in macaque visual cortex. PLoS Biol. 2011;9(4):e1000610 Epub 2011/05/03. 10.1371/journal.pbio.1000610 21532743 PMC3075230

[65] Adjamian P, Hadjipapas A, Barnes GR, Hillebrand A, Holliday IE. Induced gamma activity in primary visual cortex is related to luminance and not color contrast: an MEG study. J Vis. 2008;8(7):4 10.1167/8.7.4 19146237

[66] Swettenham JB, Muthukumaraswamy SD, Singh KD. BOLD responses in human primary visual cortex are insensitive to substantial changes in neural activity. Frontiers in Human Neuroscience. 2013;7. 10.3389/fnhum.2013.00076 PMC3593627 23482840

[67] Adjamian P, Holliday IE, Barnes GR, Hillebrand A, Hadjipapas A, Singh KD. Induced visual illusions and gamma oscillations in human primary visual cortex. European Journal of Neuroscience. 2004;20(2):587–92. 10.1111/j.1460-9568.2004.03495.x 15233769

[68] Michalareas G, Vezoli J, van Pelt S, Schoffelen J-M, Kennedy H, Fries P. Alpha-Beta and Gamma Rhythms Subserve Feedback and Feedforward Influences among Human Visual Cortical Areas. Neuron. 2016. 10.1016/j.neuron.2015.12.018 PMC4871751 26777277

[69] Lev M, Polat U. Space and time in masking and crowding. Journal of vision. 2015;15(13):10-. 10.1167/15.13.10 26381841

[70] Kreiter A, Singer W. Stimulus-dependent synchronization of neuronal responses in the visual cortex of the awake macaque monkey. The Journal of Neuroscience. 1996;16(7):2381–96. 8601818 10.1523/JNEUROSCI.16-07-02381.1996 PMC6578521

[71] Chalk M, Herrero JL, Gieselmann MA, Delicato LS, Gotthardt S, Thiele A. Attention reduces stimulus-driven gamma frequency oscillations and spike field coherence in V1. Neuron. 2010;66(1):114–25. 10.1016/j.neuron.2010.03.013 20399733 PMC2923752

[72] Bauer M, Akam T, Joseph S, Freeman E, Driver J. Does visual flicker phase at gamma frequency modulate neural signal propagation and stimulus selection? Journal of vision. 2012;12(4):5-. 10.1167/12.4.5 22505620 PMC9583753

[73] Nunez P, Srinivasan R. Scale and frequency chauvinism in brain dynamics: too much emphasis on gamma band oscillations. Brain Structure and Function. 2010;215(2):67–71. 10.1007/s00429-010-0277-6 20890614 PMC2998274

[74] Muthukumaraswamy S. High-frequency brain activity and muscle artifacts in MEG/EEG: A review and recommendations. Frontiers in Human Neuroscience. 2013;7 10.3389/fnhum.2013.00138 23596409 PMC3625857

[75] Hipp JF, Siegel M. Dissociating neuronal gamma-band activity from cranial and ocular muscle activity in EEG. Frontiers in Human Neuroscience. 2013;7:338 10.3389/fnhum.2013.00338. PMC3706727. 23847508 PMC3706727

[76] Thickbroom GW, Mastaglia FL. Presaccadic ‘spike’ potential: Investigation of topography and source. Brain Res. 1985;339(2):271–80. 10.1016/0006-8993(85)90092-7. 4027625

[77] Riemslag FCC, Van der Heijde GL, Van Dongen MMMM, Ottenhoff F. On the origin of the presaccadic spike potential. Electroencephalography and Clinical Neurophysiology. 1988;70(4):281–7. 10.1016/0013-4694(88)90046-6. 2458236

[78] Keren AS, Yuval-Greenberg S, Deouell LY. Saccadic spike potentials in gamma-band EEG: Characterization, detection and suppression. Neuroimage. 2010;49(3):2248–63. 10.1016/j.neuroimage.2009.10.057. 10.1016/j.neuroimage.2009.10.057 19874901

[79] Carl C, Açık A, König P, Engel AK, Hipp JF. The saccadic spike artifact in MEG. Neuroimage. 2012;59(2):1657–67. 10.1016/j.neuroimage.2011.09.020. 10.1016/j.neuroimage.2011.09.020 21963912

[80] Sterkin A, Yehezkel O, Bonneh YS, Norcia A, Polat U. Multi-component correlate for lateral collinear interactions in the human visual cortex. Vision Res. 2008;48(15):1641–7. Epub 2008/06/10. 10.1016/j.visres.2008.04.018 . 18538813

[81] Lamme VA, Roelfsema PR. The distinct modes of vision offered by feedforward and recurrent processing. Trends in Neurosciences. 2000;23(11):571–9. 11074267 10.1016/s0166-2236(00)01657-x

[82] Ozaktas HM, Zalevsky Z, Kutay M. The Fractional Fourier Transform with Applications in Optics and Signal Engineering. Wiley, NY; 2001.

[83] Bonneh YS, Donner TH, Sagi D, Fried M, Cooperman A, Heeger DJ, et al Motion-induced blindness and microsaccades: cause and effect. J Vis. 2010;10(14):22 Epub 2010/12/22. 10.1167/10.14.22 21172899 PMC3075454

[84] Fried M, Tsitsiashvili E, Bonneh YS, Sterkin A, Wygnanski-Jaffe T, Epstein T, et al ADHD subjects fail to suppress eye blinks and microsaccades while anticipating visual stimuli but recover with medication. Vision Res. 2014;101:62–72. Epub 2014/05/28. 10.1016/j.visres.2014.05.004 . 24863585

[85] Sterkin A, Yehezkel O, Polat U. Learning to be fast: gain accuracy with speed. Vision Res. 2012;61:115–24. Epub 2011/11/01. 10.1016/j.visres.2011.09.015 . 22037306

[86] Gieselmann M, Thiele A. Comparison of spatial integration and surround suppression characteristics in spiking activity and the local field potential in macaque V1. European Journal of Neuroscience. 2008;28(3):447–59. 10.1111/j.1460-9568.2008.06358.x 18702717

[87] Jacobs J., Manning J. R., KM J.. Response to Miller: “Broadband” vs. “high gamma” electrocorticographic signals. J Neurosci. 2010.

[88] Adini Y, Sagi D, Tsodyks M. Excitatory–inhibitory network in the visual cortex: Psychophysical evidence. Proceedings of the National Academy of Sciences. 1997;94(19):10426–31. 10.1073/pnas.94.19.10426 PMC23379 9294227

[89] Whittington MA, Cunningham MO, LeBeau FEN, Racca C, Traub RD. Multiple origins of the cortical gamma rhythm. Developmental Neurobiology. 2011;71(1):92–106. 10.1002/dneu.20814 21154913

[90] Whittington MA, Traub RD, Jefferys JGR. Synchronized oscillations in interneuron networks driven by metabotropic glutamate receptor activation. Nature. 1995;373(6515):612–5. 10.1038/373612a0 7854418

[91] Muthukumaraswamy SD, Edden RAE, Jones DK, Swettenham JB, Singh KD. Resting GABA concentration predicts peak gamma frequency and fMRI amplitude in response to visual stimulation in humans. Proceedings of the National Academy of Sciences. 2009;106(20):8356–61. 10.1073/pnas.0900728106 19416820 PMC2688873

[92] Edden RAE, Muthukumaraswamy SD, Freeman TCA, Singh KD. Orientation Discrimination Performance Is Predicted by GABA Concentration and Gamma Oscillation Frequency in Human Primary Visual Cortex. The Journal of Neuroscience. 2009;29(50):15721–6. 10.1523/JNEUROSCI.4426-09.2009 20016087 PMC6666191

[93] Hoogenboom N, Schoffelen J-M, Oostenveld R, Fries P. Visually induced gamma-band activity predicts speed of change detection in humans. Neuroimage. 2010;51(3):1162–7. 10.1016/j.neuroimage.2010.03.041. 10.1016/j.neuroimage.2010.03.041 20307670

[94] Buzsáki G, Wang X-J. Mechanisms of gamma oscillations. Annual review of neuroscience. 2012;35:203–25. 10.1146/annurev-neuro-062111-150444 22443509 PMC4049541

[95] Ozeki H, Finn IM, Schaffer ES, Miller KD, Ferster D. Inhibitory stabilization of the cortical network underlies visual surround suppression. Neuron. 2009;62(4):578–92. 10.1016/j.neuron.2009.03.028 19477158 PMC2691725

[96] Zomet A, Amiaz R, Grunhaus L, Polat U. Major depression affects perceptual filling-in. Biol Psychiatry. 2008;64(8):667–71. 10.1016/j.biopsych.2008.05.030 . 18639239

[97] Sterkin A, Yehezkel O, Zomet A, Lev M, Polat U. Pharmacological enhancement of cortical inhibition affects lateral interactions in human vision. Journal of Vision. 2009;9(8):754 10.1167/9.8.754

[98] Friston KJ, Bastos AM, Pinotsis D, Litvak V. LFP and oscillations—what do they tell us? Current opinion in neurobiology. 2015;31:1–6. 10.1016/j.conb.2014.05.004 25079053 PMC4376394

[99] Bauer M, Stenner M-P, Friston KJ, Dolan RJ. Attentional modulation of alpha/beta and gamma oscillations reflect functionally distinct processes. Journal of Neuroscience. 2014;34(48):16117–25. 10.1523/JNEUROSCI.3474-13.2014 25429152 PMC4244475

[100] Van Kerkoerle T, Self MW, Dagnino B, Gariel-Mathis M-A, Poort J, Van Der Togt C, et al Alpha and gamma oscillations characterize feedback and feedforward processing in monkey visual cortex. Proceedings of the National Academy of Sciences. 2014;111(40):14332–41. 10.1073/pnas.1402773111 PMC4210002 25205811

