# Supplementary material for: Increased gamma band activity for lateral interactions in humans
Source: PLoS One. 2017 Dec 14;12(12):e0187520. doi: 10.1371/journal.pone.0187520 (PMC5730121; doi:10.1371/journal.pone.0187520)
Supplement: S1 File — A) Spectrograms per subject for the lateral masking condition with correct responses (Lateral Hit). B) Spectrograms per subject for the linear prediction. C) Spectrograms per subject for the subtraction between Lateral Masking with correct responses (Lateral Hit) and linear prediction. D) Profiles per subject, including the values of the peaks. (PDF) [file pone.0187520.s001.pdf]

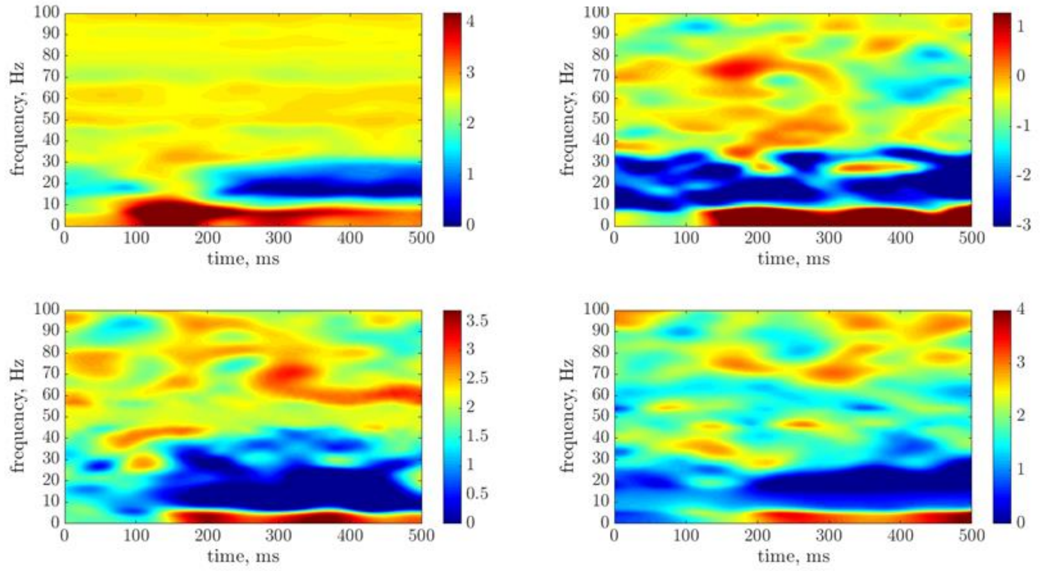

Figure A. Spectrograms per subject for the lateral masking condition with correct responses (Lateral Hit).

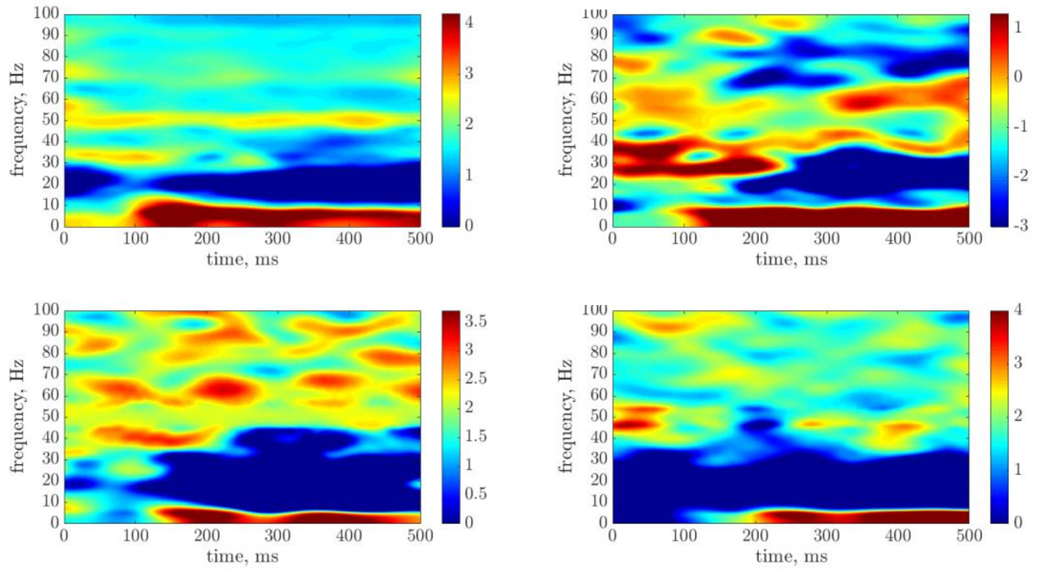

Figure B. Spectrograms per subject for the linear prediction.

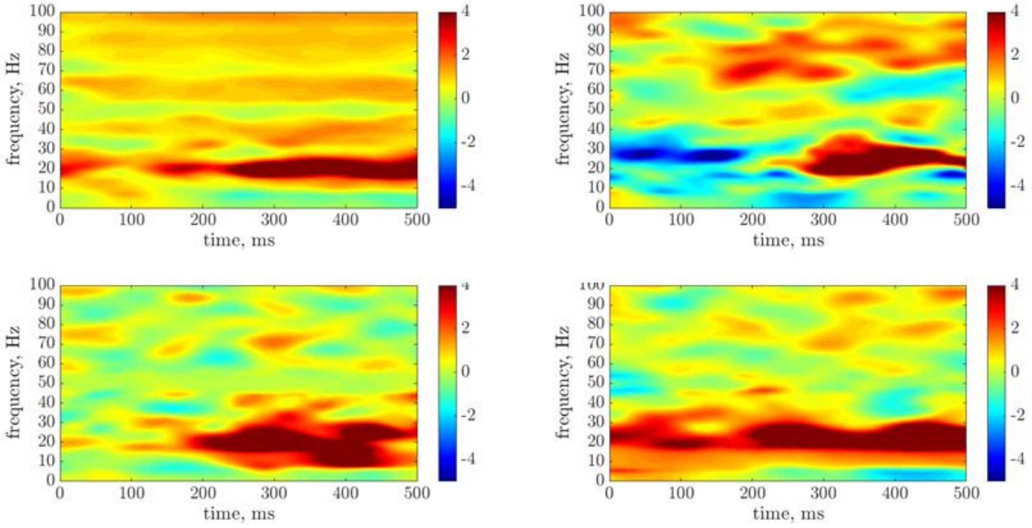

Figure C. Spectrograms per subject for the subtraction between Lateral Masking with correct responses (Lateral Hit) and linear prediction.

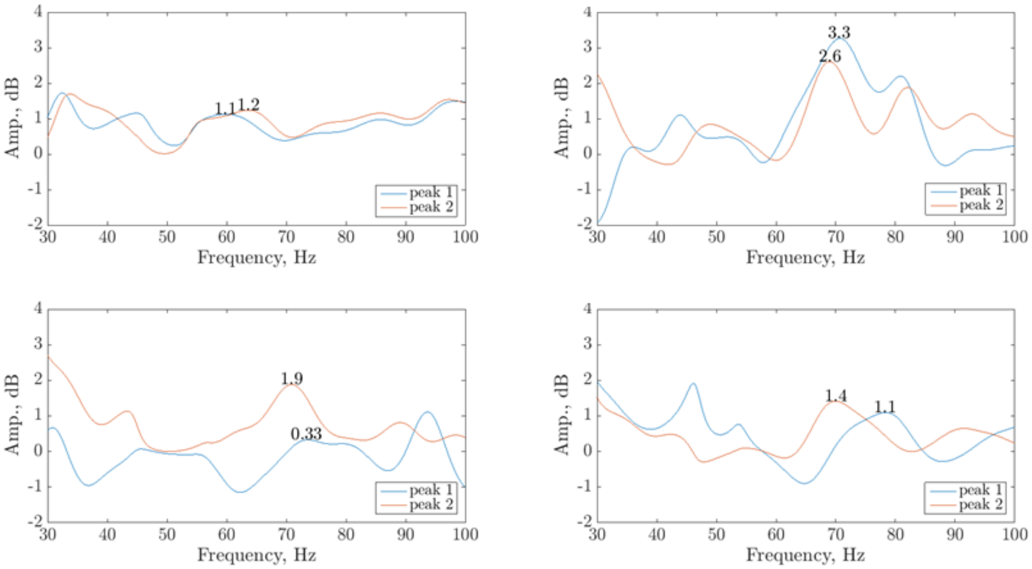

Figure D. Profiles per subject, including the values of the peaks.
